# Synthesis and Pharmacochemistry of New Pleiotropic Pyrrolyl Derivatives

**DOI:** 10.3390/molecules200916354

**Published:** 2015-09-10

**Authors:** Markella Konstantinidou, Alice Gkermani, Dimitra Hadjipavlou-Litina

**Affiliations:** Department of Pharmaceutical Chemistry, School of Pharmacy, Faculty of Health Sciences, Aristotle University of Thessaloniki, Thessaloniki 54124, Greece; E-Mails: konsmark@hotmail.com (M.K.); germanialisa@gmail.com (A.G.)

**Keywords:** chalcones, pyrroles, pleiotropic, antioxidant, anti-lipoxygenase, anti-cyclooxygenase, anti-inflammatories

## Abstract

Within the framework of our attempts to synthesize pleiotropic anti-inflammatory agents, we have synthesized some chalcones and their corresponding 3,4-pyrrolyl derivatives. Chalcones constitute a class of compounds with high biological impact. They are known for a number of biological activities, including anti-inflammatory and free radical scavenging activities. They inhibit several enzymes implicated in the inflammatory process, such as lipoxygenase, cyclooxygenase (COX) and lysozymes. The synthesized pyrroles have been studied for: (1) their *in vitro* inhibition of lipoxygenase; (2) their *in vitro* inhibition of COX; (3) their *in vitro* inhibition of lipid peroxidation; (4) their interaction with the stable, N-centered, free radical, 2,2-diphenyl-1-picrylhydrazyl (DPPH); (5) their inhibition on interleukin-6 (IL-6); (6) their anti-proteolytic activity; and (7) their *in vivo* anti-inflammatory activity using carrageenan-induced rat paw edema. Their physicochemical properties were determined to explain the biological results. Lipophilicity was experimentally determined. **2i** and **2v** were found to be promising multifunctional molecules with high antiproteolytic and anti-inflammatory activities in combination with anti-interleukin-6 activity.

## 1. Introduction

Inflammation is the natural response of the biological system to various stimuli. It is well established that excessive chronic inflammation is linked to reactive oxygen species and oxidative stress [1]. Moreover, chronic inflammation leads to pathological disorders, such as atherosclerosis, rheumatoid arthritis, neurogenerative diseases [2] and various types of cancer [3,4].

Upon appropriate stimulation of neutrophils, arachidonic acid is cleaved from membrane phospholipids through the enzymatic activity of phospholipase A2, and it is metabolized by two enzymes: cyclooxygenase (COX) and lipoxygenase leading to pro-inflammatory mediators, prostanoids and leukotrienes, respectively [5]. Regarding cyclooxygenases, COX-3 is the third and most recently discovered cyclooxygenase isozyme, the others being COX-1 and COX-2. COX-3 has been confirmed as an alternate splice variant of COX-1. Its activity appears to be selectively inhibited by acetaminophen and some other analgesic and antipyretic NSAIDs [6]. COX-1 is traditionally considered as a house-keeping enzyme, and it is responsible for the production of prostanoids and proinflammatory prostaglandins, implicated in homeostatic functions. COX-2 is the induced isoform, which catalyzes the production of prostaglandins during the inflammatory response [7]. The differential expression of the two isoforms, together with the fact that COX-1 is the main isoform expressed in the gastrointestinal track, led to the design and synthesis of selective COX-2 inhibitors as potent anti-inflammatory agents that would lack the gastro-intestinal side effects of traditional NSAIDs. Despite the initial success of selective COX-2 inhibitors, recent studies showed that they were associated with cardiovascular side effects, and some of them have been withdrawn from the world-wide market [8,9]. Furthermore, COX inhibition led to an increase of LOXs metabolites [10]. Hence, another strategy, which was followed towards more potent NSAIDs, was the design of dual inhibitors COX and LOX, which would have simultaneously the advantage of blocking both enzymes in the arachidonic acid cascade [11].

COX-1 and COX-2 possess distinct roles in neuroinflammation. Taking into account the differential expression of the two isoforms in central nervous system (CNS), COX-1 selective inhibition has lately emerged as a new therapeutic target for CNS inflammatory diseases [12,13,14].

Nowadays, there is great interest in the use of pleiotropic drugs for the treatment of complex diseases, in which more than one target is implicated. In the last decade, we have designed and synthesized several potent lipoxygenase inhibitors, antioxidants and anti-inflammatories guided by our Quantitative–Structure Activity Relationships (QSAR) results. Since inflammation is a complex phenomenon in which several different factors are implicated, pleiotropic agents will offer additional benefits. In light of this, we designed a number of 3,4-substituted pyrroles. The design principle was aimed at combining the synergistic property of biological potent chalcones to get new heterocyclic entities: pyrrolyl derivatives that might act as effective pleiotropic bioactive agents. Yet another objective of the study was to evaluate the effect of steric and electronic parameters on anti-inflammatory activities and to optimize the activity through systematic modification of the pyrrole 3,4-substituents.

Chalcones or 1,3-diaryl-2-propen-1-ones are α,β-unsaturated ketones and have a large number of different biological activities. These compounds occur naturally or they are readily synthesized. In the literature, derivatives bearing the chalcone scaffold present antibacterial [15], antifungal [16], analgesic [17], anti-inflammatory [18], anticancer [19], antioxidant [20], immunosuppressive [21], gastroprotective [22], antiviral [23] and antileishmanial [24] activities.

Pyrroles exists both in natural and synthetic products, and their ability to form chelate complexes with metals explains their significance in biological systems. Characteristic examples of tetra-pyrrole structures are heme, chlorophyll and vitamin B_12_. Pyrrole derivatives with various actions are reported in the literature: antimalarial [25], antimicrobial [26], anti-inflammatory [27], antioxidant [28], HIV fusion inhibitors [29], anticancer [30,31,32], inhibitors of dipeptidyl peptidase IV [33], selective potassium-competitive acid blockers [34] and inhibitors of HMG-CoA reductase [35]. Diarylpyrrolizines have been investigated as COX and LOX inhibitors [36]. Pyrroles with known anti-inflammatory activity, e.g., ketorolac and tolmetin, are characterized by an aroyl pyrrole moiety. A carbonyl group in the vicinal diaryl substitution is the structural feature in the design of COX-2 inhibitors. Earlier publications refer to the influence of vicinal aryl, aroyl and carbonyl unit combinations on enzyme inhibitory activity within the synthesized pyrroles [36].

From a synthetic point of view, chalcones are useful synthons for heterocyclic compounds, since the α,β-unsaturated moiety can be easily exploited towards the synthesis of heterocycles with numerous reagents. With this in mind, we synthesized and used a number of chalcones, which presented antioxidant and anti-lipoxygenase activities as precursors for the synthesis of our pyrrolyl derivatives.

## 2. Results and Discussion

### 2.1. Chemistry

**Scheme 1:**
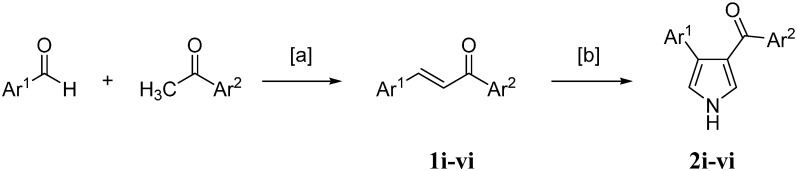
Derivatives via a van Leusen reaction.

The synthesis of chalcones was accomplished by a Claisen–Schmidt condensation between a substituted aromatic ketone and a suitable substituted aromatic aldehyde [37], as depicted in Scheme 1. Chalcones were used as precursors for the synthesis of aryl-aroyl-substituted pyrrolyl derivatives via a van Leusen reaction [38] with tosylmethyl isocyanide (TosMIC) to give 3-aroyl-4-arylpyrroles in an intermolecular cyclization. The ring carbons C2 and C5 originate from TosMIC, whereas C3 and C4 are derived from the chalcones that act as Michael acceptors. The used chalcones present a variation of interesting preliminary anti-inflammatory biological activities and physicochemical properties, and thus, we have chosen them as promising synthons (Table 1).

(a) MeOH, KOH 15% *w*/*v*, ultrasound bath, yields 57%–78%; (b) TosMIC, NaH, DMSO/Et_2_O, ice-bath, yields 17%–98%.

**Table 1:** Chemical structures, physicochemical and reaction data of chalcones **1i**–**1vi**.

No.	Ar^1^	Ar^2^	R_f_^#^	m.p. (°C)	Yield (%)
**1i**	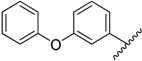	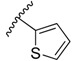	0.7	48–50	57
**1ii** [39]	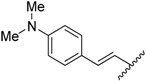	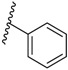	0.6	113–115	75
**1iii**	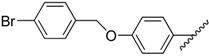	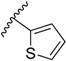	0.8	141–143	88
**1iv** [40]	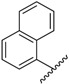	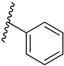	0.8	48–50	78
**1v** [41]	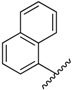	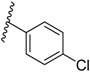	0.7	48–50	86
**1vi** [42]	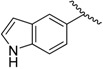	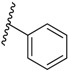	0.5	48–50	66

^#^ Dichloromethane.

The structural characteristics of the known synthesized chalcones, Compounds **1ii** [39], **1iv** [40], **1v** [41], **1vi** [42], were in agreement with the literature. The structure of the new **1i** and **1iii** compounds (with yields from 57%–88%) have been identified by their physicochemical and spectroscopic data. The final products were obtained in good yields (30%–96%), with the only exception being Compound **2iv**, which was obtained in a lower yield (17%). The final products were recrystallized from cold ethyl acetate. IR, ^1^H-NMR, ^13^C-NMR and elemental analysis supported the proposed structures of the synthesized pyrrolyl derivatives. The physical data of the synthesized compounds are given in detail in the Experimental Section.

### 2.2. Physicochemical Studies

#### 2.2.1. Determination of Lipophilicity

Since lipophilicity is a significant physicochemical property determining the absorption, distribution, metabolism and excretion properties (ADME) of drugs, we attempted to determine experimentally the lipophilicity as R_M_ values with reverse-phase thin layer chromatography (RPTLC) [43]. Lipophilicity was also theoretically calculated as Clog *P* values [44,45]. According to the calculated Clog *P* values, the most lipophilic compounds were **2iii** (6.37) and **2v** (6.04). However, this observation was not supported by the R_M_ values, since Compound **2iii** presented very low lipophilicity (−0.061). Furthermore, the lipophilicity of Compound **2v** could not be determined experimentally under the reported conditions. Attempts to correlate Clog *P* and R_M_ values failed. From our results (Table 2), it can be concluded that R_M_ values could not be used as a successful relative measure of the overall lipophilic/hydrophilic properties of these molecules. Tentatively, this can be attributed to the different nature of the hydrophilic and lipophilic phases in the two systems. Clog *P* refers to *n*-octanol/water, whereas R_M_ in this case refers to methanol/water. Moreover, it is likely that pyrrole derivatives form hydrogen bonds within the hydrophilic phase.

**Table 2:** Chemical structures, physicochemical and reaction data of pyrrole derivatives **2i**–**2vi**.

No.	Ar^1^	Ar^2^	Clog *P*	R_M_	R_f_^#^	m.p. (°C)	Yields (%)
**2i**	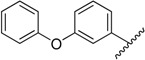	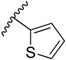	5.90	0.091	0.7	165–166	49
**2ii**	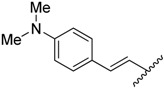	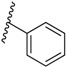	5.05	0.073	0.6	219–220	96
**2iii**	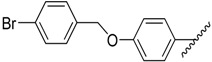	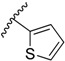	6.37	−0.061	0.9	233–235	43
**2iv**	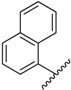	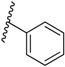	5.26	−0.149	0.8	270–272	17
**2v**	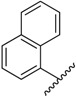	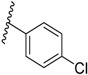	6.04	Nd	0.9	285–286	30
**2vi**	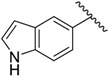	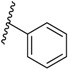	4.07	−0.685	0.7	247–248	32

Nd: not determined under these conditions; ^#^ chloroform/ethyl acetate 2:1.

#### 2.2.2. Theoretical Calculation of Physicochemical Properties

The physicochemical properties were determined with the program Spartan v.5.1.3. (Wavefunction Inc., Irvine, CA, USA) (Table 3) in the conformation of minimum energy. The values of overall molecular refractivity (CMR) and molar refractivity of substituents (MR) (Table 4) were predicted with the C-QSAR program of Biobyte [45].

**Table 3:** Theoretical calculation of the properties associated with energy and charge distribution for pyrrole derivatives **2i**–**2vi**.

No.	*E*_(HOMO)_ (eV)	*E*_(LUMO)_ (eV)	Δ*E*_(HOMO-LUMO)_ (eV)	SM_2_ (eV)	Elpot__MIN_ (eV)	Elpot__MAX_ (eV)	Dipole (D)
**2i**	−7.95	2.42	10.37	2.12	−2.16	3.12	6.06
**2ii**	−7.12	2.77	9.89	2.02	−2.30	2.86	4.33
**2iii**	−7.78	2.45	10.23	0.75	−2.70	3.16	6.37
**2iv**	−7.21	2.41	9.62	2.53	−2.26	2.95	4.13
**2v**	−7.29	2.31	9.6	2.43	−2.13	3.01	4.99
**2vi**	−7.50	-1.91	5.59	9.85	−2.12	1.63	4.14

**Table 4:** Theoretical calculation of molar refractivity, surface area and volume for pyrrole derivatives **2i**–**2vi**.

No	CMR	MR (Ar^1^)	MR (Ar^2^)	Surface Area (Å^2^)	Volume (Å^3^)
**2i**	9.723	5.250	2.395	381.77	376.32
**2ii**	9.902	4.860	2.586	351.86	343.56
**2iii**	11.266	6.491	2.395	430.45	421.74
**2iv**	9.328	4.274	2.586	345.60	341.68
**2v**	10.404	4.274	3.077	363.97	359.95
**2vi**	8.611	3.614	2.586	317.63	314.83

### 2.3. Biological Evaluation of Antioxidant and Anti-Inflammatory Activity

In the present study, six new pyrrole derivatives were synthesized as pleiotropic agents against several targets implicated in inflammation. As starting materials, we used a number of promising chalcones, which have been tested as antioxidants/anti-inflammatories.

Taking into account the multifactorial character of oxidative stress and inflammation, we decided to evaluate *in vitro* the chalcones, as well as the pyrrolyl derivatives with regard to their antioxidant ability, as well as to their ability to inhibit enzymes implicated in the inflammation process, such as: (i) LOX; (ii) COX-2; (iii) interleukin-6 and (iv) trypsin.

Many non-steroidal anti-inflammatory drugs have been reported to act either as inhibitors of free radical production or as radical scavengers [46]. Consequently, lipid peroxidation inhibitors [47] could be expected to offer protection in inflammation and to lead to potentially effective agents.

We used several different assays to measure the antioxidant activity *in vitro*: (1) the interaction with the stable free radical 2,2-diphenyl-1-picrylhydrazyl (DPPH); (2) the non-enzymatic method of superoxide radical scavenging activity; and (3) the inhibition of heme-dependent lipid peroxidation of linoleic acid. The antioxidant ability of a compound should be tested in a variety of milieus to clarify the impact of factors, such as solubility or steric hindrance, which may be of overriding importance in some cases.

2,2-Diphenyl-1-picrylhydrazyl (DPPH) is a lipophilic, free, stable radical, which absorbs at 517 nm (violet color). Upon reduction, decolorization is observed that is stoichiometric to the number of received electrons and is representative of the antioxidant ability of compounds. The interaction/reducing activity (RA) of the examined compounds with the stable free radical DPPH is shown in Table 5. For the sake of comparison, RA % values for nordihydroguaiaretic acid (NDGA) were included in the table. This interaction, which indicates their radical scavenging ability in an iron-free system, was measured at 100 and 1000 μM after 20 and 60 min, whereas the final concentration of DPPH was stable at 50 μΜ. With the exception of **1i** and **1ii**, which interact with DPPH, all of the other chalcones did not have any effect.

Considering the pyrrolyl derivatives, the results, given in Table 6, indicate no action or very low activity under these experimental conditions. It is possible that the tested compounds failed to interact with the DPPH radical owing to steric factors. Compound **2ii** presented remarkable reducing activity at both concentrations, which was higher than that of the reference compound NDGA. Compound **2ii** was additionally tested at a 10 μΜ concentration and presented 35% and 40% interaction at 20 and 60 min, respectively. By examining the structures of the compounds, it is likely that the antioxidant ability of Compound **2ii** correlated with the presence of the double bond, a feature that is missing from any other pyrrolyl derivative.

**Table 5:** Reducing ability measured as the interaction with DPPH (reducing activity % (RA %)); inhibition of rat paw edema induced by carrageenan (CPE %).

Comp.	RA % (100 μΜ)		RA % (1000 μΜ)		CPE % (0.01 mmol/kg Body Weight)
	20 min	60 min	20 min	60 min	
**1i**	43	2	5	na	41 ^c^
**1ii**	31	13	38	42	69 ^d^
**1iii**	na	na	na	na	86 ^d^
**1iv**	na	na	na	na	40 ^d^
**1v**	na	na	na	na	44 ^d^
**1vi**	na	na	na	na	42 ^c^
**NDGA**	81	83	96	96	^−^
**Indomethacin**					53 ^d^

na: no activity under the reported experimental conditions; ^c^ *p* < 0.05; ^d^ *p* < 0.01 performed with Student’s *t*-test.

**Table 6:** Reducing ability measured as interaction with DPPH (RA %); superoxide radical scavenging activity (O_2_^•−^ %); inhibition of heme-dependent lipid peroxidation of linoleic acid (LP, IC_50_ values).

Comp.	RA % (100 μΜ)		RA % (1000 μΜ)		O_2_^•−^ % (100 μΜ)	LP IC_50_ (μΜ)
	20 min	60 min	20 min	60 min		
**2i**	na	na	na	na	57	40
2ii *	86	88	99	96	na	na
**2iii**	na	na	4	5	57	100
**2iv**	na	na	8	10	76	72
**2v**	na	na	na	na	na	150
**2vi**	na	na	na	na	86	na
**NDGA**	81	83	95.6	^−^	^−^	^−^
**Tolmetin**	3	3	4	9	^−^	^−^
**Caffeic acid**	^−^	^−^	^−^	^−^	86.1	6

* Thirty five percent (20 min) and 40% (60 min) at 10 μΜ, na: no activity under the reported experimental conditions.

Superoxide anion radical is an active form of oxygen, and it is the product of the one-electron reduction of molar oxygen. Herein, superoxide radicals were formed by mixing phenazine methosulfate (PMS) with NADH and air-oxygen. The production of radicals was estimated with the nitroblue-tetrazolium method (NBT). From the tested pyrroles, Compounds **2i** and **2iii** were equipotent (57%) scavengers. The most active compounds were **2iv** and **2vi** with 76 and 87%, respectively. Worth noting was that derivative **2vi** presented comparable activity to caffeic acid, a known reference compound. Ar^2^ substitution for the two most active derivatives was a phenyl group, whereas for Compound **2iv**, Ar^1^ was a naphthyl group, and for Compound **2vi**, Ar^1^ was an indolyl group. It should to be mentioned that Compound **2v** (Ar^1^ = naphthyl, Ar^2^ = 4-chlorophenyl) was inactive under the reported experimental conditions. It seems that lipophilicity does not significantly influence the superoxide anion scavenging activity. In contrast, the dipole moment appeared to be important. The values of the dipole, as calculated by the program Spartan v.5.1.3. (Wavefunction Inc.), were similar for Compounds **2iv** and **2vi** (4.13 and 4.14 D, respectively), whereas for Compound **2v**, the value of the dipole was 4.99 D. Hence, the increase of dipole values correlated with a reduction in the ability of compounds to scavenge superoxide anion radicals.

The anti-lipid peroxidation activity was determined through the inhibition of heme-dependent lipid peroxidation. The peroxidation of sodium linoleate is induced from a mixture of heme and hydrogen peroxide, which produces free radicals from the side chains of amino acids. The assay is performed at pH 7.4 with a phosphate buffer, and the products of peroxidation are identified with the thiobarbituric acid method (TBA) [48]. In particular, malonic dialdehyde, a final product of lipid peroxidation, reacts with TBA and forms a colored product, whose absorbance is measured at 535 nm. Pyrrolyl derivatives presented moderate inhibitory activity, with the exception of **2i** (IC_50_ = 40 μΜ). Compounds **2ii** and **2vi** were inactive under these experimental conditions. In general, pyrrolyl derivatives were less active than the reference compound: caffeic acid. Compounds **2ii** and **2vi** with low Clog *P* values are weak inhibitors of lipid peroxidation. It seems that the increase of volume of Ar^1^, measured as molar refractivity MR (Ar^1^), enhances IC_50_ values and reduces the inhibitory activity (**2i** and **2iii**; and **2ii**, **2iv** and **2vi**). For derivatives **2iv** and **2v**, the presence of an electron-acceptor, e.g., a chloro-substituent on the phenyl ring (Ar^2^), diminishes the anti-lipid peroxidation activity.

Taking into account the multifactorial character of oxidative stress and inflammation, the pyrrolyl derivatives were evaluated for their anti-inflammatory activity. The following biological assays were performed *in vitro*: (1) inhibition of soybean lipoxygenase; (2) inhibition of COX-1; (3) inhibition of COX-2; (4) inhibition of IL-6 and (5) inhibition of the proteolytic activity of trypsin. For the anti-inflammatory *in vivo* assay, we used rat paw edema induced by carrageenan.

Lipoxygenase catalyzes the first two steps in the metabolism of arachidonic acid to leukotrienes. LTB4 generation is important in the pathogenesis of neutrophil-mediated inflammatory diseases [49] with a marked relation to the severity of cardiovascular diseases, asthma and cancer. We evaluated the synthesized pyrroles for their ability to inhibit soybean LOX by the UV absorbance-based enzyme assay [50]. All pyrroles presented very low or no action under these experimental conditions (Table 7). The main reason for the lack of inhibitory activity was attributed to be the bulk of molecules. We succeeded at determining the IC_50_ value only for Compound **2i**. The inhibition of COX-2, as a peroxidase, was determined by a colorimetric assay with the oxidation of *N*,*N*,*N′*,*N′*-tetramethyl-*p*-phenylenediamine (TMPD). Compounds **2iii**, **2iv** and **2vi** were inactive under the experimental conditions (Table 7). The most potent COX-2 inhibitor at a 100 μΜ concentration was Compound **2v** (Ar^1^ = naphthyl, Ar^2^ = *p*-chlorophenyl), followed by **2i**. The structurally-related **2iv** (Ar^1^ = naphthyl, Ar^2^ = phenyl) was inactive. It is possible that electron-acceptor substituents influence the activity. Indomethacin, which was included in the study for the sake of comparison, presented 95% inhibition at 100 μΜ.

The assay used for the inhibition of IL-6 is based on an immunometric double antibody method (sandwich technique). In this assay, Compounds **2i** and **2v** were studied, which were previously proven to be the most potent COX-2 inhibitors. Both compounds gave satisfactory biological responses, both in high and in low concentrations of IL-6 (Table 8). It should to be mentioned that both compounds have been tested at 10 μΜ, a concentration significantly lower than the concentration used in the COX-2 assay. Both Compounds **2i** and **2v** exhibited better results compared to indomethacin.

Preliminary results (data not given) support that Compound **2v** is a double COX-1 (75% at concentration of 100 μΜ) and COX-2 inhibitor, whereas Compound **2iii** is a selective COX-1 inhibitor (75% at concentration of 100 μΜ), without any activity against COX-2. More experiments are in progress to delineate accurately the inhibition profile.

**Table 7:** Inhibition of soybean lipoxygenase (LOX IC_50_ μΜ or % at 100 μM); percent inhibition of cycloxygenase-2 (COX-2 % at 100 μΜ); inhibition of trypsin (TP IC_50_ μΜ); inhibition of rat paw edema induced by carrageenan (CPE %).

Comp.	LOX IC_50_ (μΜ)/% (100 μM)	COX-2 % (100 μM)	TP IC_50_ (μΜ)	CPE % (0.01 mmol/kg Body Weight) or ED_50_ mmol/kg Body Weight
**2i**	1000 μΜ	67.2	60	0.061 ^c^
**2ii**	na	47	na	89% ^d^
**2iii**	na	na	9	0.0525(89%) ^c^
**2iv**	10%	na	65	0.064 (75%) ^c^
**2v**	32%	81	8	0.063 (78%) ^c^
**NDGA**	43 μΜ	nt	nt	nt
**Tolmetin**	190 μΜ	nt	nt	76%
**Indomethacin**	^−^	95	nt	53% ^d^
**Salicylic acid**	^−^	-	100	nt

na: no activity under these experimental conditions, nt: not tested; ^c^ *p* < 0.05; ^d^ *p* < 0.01 performed with Student’s *t*-test.

**Table 8:** Percent inhibition of interleukin-6 (IL-6).

IL-6 pg/mL	Comp. 2i (10 μΜ) % Inhibition	Comp. 2v (10 μΜ) % Inhibition	IMA (10 μΜ) % Inhibition
1500	99	100	100
750.0	98	99	na
375.0	99	99	na
187.5	99	na	na
93.7	na	na	na
46.8	na	na	na
23.4	na	na	na

na: no activity under these experimental conditions; IMA: indomethacin.

Trypsin is an enzyme with an active role in inflammation. Thus, we studied the anti-proteolytic ability of the pyrrole derivatives, using salicylic acid as a reference compound. Compounds **2i** and **2iv** presented IC_50_ values of 60 and 65 μΜ, respectively, whereas Compounds **2iii** and **2v** were much more potent, with IC_50_ values of 9 and 8 μΜ, respectively. Comparing Compounds **2iv** and **2v**, the insertion of an electron-acceptor substituent (Cl) on the phenyl ring (substituent Ar^2^) and the simultaneous increase of the volume of substituent Ar^2^ (expressed as molar refractivity) led to an enhancement of the anti-proteolytic activity.

**Scheme 2:**
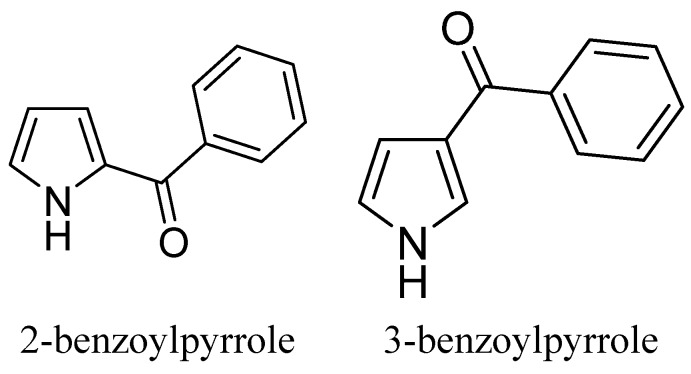
Structures of mono-substituted pyrrole derivatives.

The pyrrolyl derivatives, as well as their precursor chalcones were tested *in vivo* for the inhibition of rat paw edema induced by carrageenan. Chalcones **1iii** and **1ii** highly reduced the rat paw edema at 0.01 mmol/kg body weight, whereas **1i**, **1iv**, **1v** and **1vi** presented lower and almost equal response. It should to be noted that the most potent chalcones **1ii** and **1iii** are the precursors of the two most active pyrrolyl derivatives. All pyrrolyl derivatives at 0.01 mmol/kg body weight showed anti-inflammatory activity comparable or higher than indomethacin, which was the reference compound, and tolmetin, which was included in the study as a pyrrolyl NSAID compound. Compound **2ii** showed an inhibition of 89%. The two naphthyl-substituted derivatives **2iv** and **2v** presented almost equivalent inhibition (75% and 78%, respectively), indicating that the bulky substituents significantly influence the biological activity. For Compounds **2i**, **2iii**, **2iv** and **2v**, we determined their ED_50_ values. Considering these results, **2iii** with ED_50_ = 0.052 mmol/kg body weight was the most potent anti-inflammatory agent. The finding supports the primary idea of this design. Two mono-substituted pyrroles (Scheme 2), 2-benzoylpyrrole and 3-benzoylpyrrole, which, at a dose of 0.44 mmol/kg, showed 34% and 43% inhibition of carrageenan-induced rat paw edema, respectively, were previously studied for their ability to inhibit carrageenan-induced edema [51].

The insertion of the substituent in position 3 rather than 2 favors the inhibitory activity. Although the role of the physicochemical properties is not entirely clear for the biological results, a comparison of *in vitro* and *in vivo* results underlines a correlation between them and, in particular, among the results from the inhibition of COX-2, trypsin, IL-6 and antioxidant activity. Comparing the data from mono- and bis-substituted pyrrolyl derivatives, a second bulky substituent in position 4 significantly improved the anti-inflammatory activity *in vivo*. The absence of a free carboxylic group in the synthesized derivatives is considered to be important owing to its implication in adverse side effects. Such adverse side effects may occur with “classic” non-steroid anti-inflammatory drugs, in which tolmetin belongs.

## 3. Experimental Section

### 3.1. Materials and Instruments

All chemicals, solvents, chemical and biochemical reagents were of analytical grade and purchased from commercial sources (Merck, Merck KGaA, Darmstadt, Germany, Fluka Sigma-Aldrich Laborchemikalien GmbH, Hannover, Germany, Alfa Aesar, Karlsruhe, Germany and Sigma, St. Louis, MO, USA).

For the biological assays, soybean lipoxygenase, pancreatic bovine trypsin, sodium linoleate, free radical DPPH, PMS, NADH, nitroblue tetrazolium, caffeic acid, heme, H_2_O_2_, trichloroacetic acid, acetic acid, salicylic acid (SA), nordihydroguaiaretic acid (NDGA), *N*,*N*,*N′*,*N′*-tetramethyl-*p*-phenylenediamine (TMPD) and arachidonic acid were obtained from Sigma Chemical, Co. (St. Louis, MO, USA). Carrageenan, type K, was commercially available. Cycloxygenase-2 (COX-2) and interleukin-6 (IL-6) EIA Kit, Item No. 583371 were purchased from Cayman. Trolox was obtained from Aldrich Chemical Co. (Milwaukee, WI, USA). All starting materials were obtained from commercial sources, and they were used without further purification.

Melting points (uncorrected) were determined on an MEL-Temp II (Lab Devices, Holliston, MA, USA). For the *in vitro* tests, UV-VIS spectra were obtained on a Perkin-Elmer 554 double-beam spectrophotometer (Perkin-Elmer Corporation Ltd., Lane Beaconsfield, Bucks, UK). Infrared spectra (film as Nujol mulls or KBr pellets) were recorded with a Perkin-Elmer 597 spectrophotometer (Perkin-Elmer Corporation Ltd.). The ^1^H-NMR spectra were recorded at 300 MHz on a Bruker AM-300 spectrometer (Bruker Analytische Messtechnik GmbH, Rheinstetten, Germany) in CDCl_3_ or DMSO using tetramethylsilane as an internal standard, unless otherwise stated. ^13^C-NMR spectra were obtained at 75.5 MHz on a Bruker AM-300 spectrometer in CDCl_3_ or DMSO solutions with tetramethylsilane as the internal reference, unless otherwise stated. Chemical shifts are expressed in δ (ppm) and coupling constants *J* in Hz. Mass spectra were determined on a LC-MS 2010 EV Shimadzu (Shimadzu Scientific Instruments, Inc., Columbia, MA, USA), using MeOH as a solvent. Elemental analyses for C and H gave values acceptably close to the theoretical values (±0.4%) in a Perkin-Elmer 240B CHN analyzer (Perkin-Elmer Corporation Ltd.). Reactions were monitored by thin layer chromatography on 5554 F_254_ silica gel/TLC cards, Merck and Fluka Chemie GmbH Buchs, Steinheim, Switzerland. For preparative thin layer chromatography (PLC), silica gel 60 F_254_, plates 2 mm, Merck KGaA ICH078057, were used. For the experimental determination of the lipophilicity reverse phase thin layer chromatography (RPTLC), TLC-silica gel 60 F_254_ DC Kieselgel, Merck (20 × 20 cm) plates were used.

### 3.2. Chemistry General Procedure

#### 3.2.1. Chemistry General Procedure for Chalcones

A modified Claisen–Schmidt condensation was followed. Equimolar amounts of a suitable substituted acetophenone (10 mmol) and of a suitable substituted aromatic aldehyde (10 mmol) were used. Each reagent was dissolved in methanol, and the total volume of the used solvent was 20 mL. The methanolic solution of the substituted acetophenone was slowly added under stirring in the methanolic solution of the aldehyde, followed by the addition of 5 mL of potassium hydroxide (15% *w*/*v*). The reaction was carried out in an ultrasound bath and has been monitored by thin-layer chromatography. The reaction mixture was cooled by an ice bath and was acidified with an aqueous solution of hydrochloric acid (10%). The precipitate was filtered off and washed repeatedly with water. In case no precipitate was formed, the mixture was extracted with 3 × 40 mL of dichloromethane or chloroform; the organic layer was collected, dried over sodium sulfate, evaporated to dryness and was recrystallized with methanol. Chalcones **1ii**, **1iv**–**vi** are referred to in the literature.

*3-(3-Phenoxyphenyl)-1-(thien-2-yl)prop-2-en-1-one* (**1i**): the modified general method was followed using 3-phenoxybenzaldehyde and 2-acetylthiophene. IR (Nujol) (ν cm^−1^): 3100m (Ar-H), 1650s (C=O), 1580m (C=C). ^1^H-NMR (300 MHz, CDCl_3_): δ (ppm) 7.03–7.44 (m, 10H, 9 × Ph H, 1 × COCH=, 1 × thienyl Η), 7.86–7.68 (m, 4H, 3 × thienyl H and 1 × CH=). ^13^C-NMR (75.5 MHz, CDCl_3_): δ (ppm) 172.9, 154.6, 153.2, 145.4, 143.3, 136.6, 134.0, 131.9, 130.3, 129.9, 128.2, 123.7, 123.5, 122.3, 120.8, 119.1, 118.1, 77.4, 77.0, 76.6. Anal. C, H. (C_19_H_14_O_2_S); Calc. % C: 74.18, H: 4.60; found % C: 74.17, H: 4.61.

*5-[4-(Dimethylamino)phenyl]-1-phenylpenta-2,4-dien-1-one* (**1ii**): The modified synthetic method was used. The analytical data, as well as the spectral analysis were in agreement with those given in the literature [39].

*3-{4-[(4-Bromobenzyl)oxy]phenyl}-1-(thien-2-yl)prop-2-en-1-one* (**1iii**): the modified general method was followed using 4-[(4-bromobenzyl)oxy]benzaldehyde and 2-acetylthiophene. IR (Nujol) (ν cm^−1^): 3100m (Ar-H), 1680s (C=O), 1580m (C=C). ^1^H-NMR (300 MHz, CDCl_3_): δ (ppm) 5.07-5.11 (br, 2H, OCH_2_), 6.97–7.32 (m, 6H, 4 × Ph H, 1 × γ*-*thienyl-Η, 1 × H COCH=), 7.51–7.85 (m, 7H, 4 × Ph H, 1 × β*-*thienyl-H, 1 × δ-thienyl-H, 1 × H CH=CH). ^13^C-NMR: (75.5 MHz, CDCl_3_): δ (ppm) 180.7, 160.6, 157.7, 143.7, 142.2, 135.5, 133.5, 131.8, 131.5, 130.3, 129.0, 128.5, 128.2, 128.0, 127.3, 123.6, 119.7, 115.3, 109.3. [Μ^+^] 399 (11), 111 (46), 83 (7); Anal. C, H. (C_20_H_15_O_2_BrS); Calc. % C: 60.15, H: 3.75; found % C: 59.81, H: 3.85.

*3-(Naphth-1-yl)-1-phenylprop-2-en-1-one* (**1iv**): The modified synthetic method was used. The analytical data, as well as the spectral analysis were in agreement with those given in literature [40].

*3-(Naphth-1-yl)-1-(4-chloro)phenylprop-2-en-1-one* (**1v**): The modified synthetic method was used. The analytical data, as well as the spectral analysis were in agreement with those given in the literature [41].

*3-(1H-Indol-5-yl)-1-phenylprop-2-en-1-one* (**1vi**): The modified synthetic method was used. The analytical data, as well as the spectral analysis were in agreement with those given in the literature [42].

#### 3.2.2. General Method for the Synthesis of Pyrrolyl Derivatives

The reaction was carried out according to a modified literature method [38]. All glassware and all used solvents were dry. Equimolar quantities of the appropriate chalcones and TosMIC were dissolved in a mixture of dry DMSO and dry diethyl ether (1:2.5). The reaction mixture was added dropwise under stirring into a suspension of sodium hydride (30 equiv. of the chalcone) in dry diethyl ether, to keep the hydrogen formation under control. The reaction was carried out in an ice bath for 24 h. In continuation, the mixture was poured in 500 mL ice water. The pH of the solution was strongly basic. The organic phase was separated. The aquatic phase was extracted three times with ethyl acetate, dried over sodium sulfate and concentrated under vacuum. The residue was treated with cold methanol and recrystallized (cold ethyl acetate) to give the pure product.

*[4-(3-Phenoxyphenyl)-1H-pyrrol-3-yl](thien-2-yl)methanone* (**2i**): The above mentioned general method was followed using chalcone **1i**. IR (Nujol) (ν cm^−1^): 3350m (N-H), 1640s (C=O), 1580m (C=C). ^1^H-NMR (300 MHz, CDCl_3_): δ (ppm) 8.00–8.07 (m, 1H, thienyl H), 7.58–7.62 (m, 1H, 1 × thienyl H), 7.37–7.39 (m, 3H, 3 × Ph H), 7.29–7.32 (m, 1H, 1 × Ph H), 7.03–7.27 (m, 6H, 3 × Ph H, 2 × pyrrolyl H, 1 × thienyl H), 6.84–6.99 (m, 3H, 3 × Ph H). ^13^C-NMR (75.5 MHz, CDCl_3_): δ (ppm) 165.0, 133.1, 132.6, 129.6, 129.0, 127.5, 125.0, 123.9, 122.9, 119.3, 118.6, 118.5, 117.0. Anal. C, H, N. (C_21_H_15_NO_2_S); Calc. % C: 72.39, H: 4.34, N: 4.04; found % C: 72.22, H: 4.57, N: 3.73.

*(E)-{4-[4-(Dimethylamino)styryl]-1H-pyrrol-3-yl}(phenyl)methanone* (**2ii**): The general method was followed using chalcone **1ii**. IR (Nujol) (ν cm^−1^): 3295m (N-H), 1681s (C=O), 1614m (C=C). ^1^H-NMR (300 MHz, CDCl_3_): δ (ppm) 7.80–7.82 (m, 2H, 2 × Ph H), 7.37–7.58 (m, 5H, 5 × Ph H), 7.10–7.12 (m, 2H, 2 × vinyl H, 6.75–6.89 (m, 4H, 2 × Ph H, 2 × pyrrolyl H), 2.96 (s, 6H, 2 × methyl H). ^13^C-NMR (75.5 MHz, CDCl_3_): δ (ppm) 164.0, 131.1, 129.0, 128.0, 127.8, 127.3, 127.0, 116.0, 113.0, 22.0. Anal. C, H, N. (C_21_H_20_N_2_O); Calc. % C: 79.72, H: 6.37, N: 8.85; found % C: 80.05, H: 6.37, N: 8.73.

*(4-{4-[(4-Bromobenzyl)oxy]phenyl}-1H-pyrrol-3-yl)(thien-2-yl)methanone* (**2iii**): The general method was followed using chalcone **1iii**. IR (Nujol) (ν cm^−1^): 3500–3450br s (N-H), 1710s (C=O), 1580m (C=C). ^1^H-NMR (300 MHz, CDCl_3_): δ (ppm) 8.51–8.53 (br, 1H, 1 × thienyl Η), 7.58–7.68 (m, 3H, 2 × Ph H, 1 × thienyl H), 7.49–7.52 (m, 2H, 2 × Ph H), 7.29–7.40 (m, 3H, 2 × Ph H, 1 × thienyl H), 7.07–7.11 (br, 1H, 1 × pyrrolyl H), 6.88–7.05 (m, 4H, 3 × Ph H, 1 × pyrrolyl H), 5.00 (s, 2H, 2 × CH_2_O). ^13^C-NMR (75.5 MHz, CDCl_3_): δ (ppm) 183.0, 133.0, 132.0, 131.0, 130.0, 129.0, 127.0, 125.0, 118.0, 111.0, 77.0. Anal. C, H, N. (C_22_H_16_BrNO_2_S); Calc. % C: 60.28, H: 3.68, N: 3.19; found % C: 60.15, H: 3.93, N: 3.10.

*[4-(Naphth-1-yl)-1H-pyrrol-3-yl](phenyl)methanone* (**2iv**): The general method was followed using chalcone **1iv**. IR (Nujol) (ν cm^−1^): 3450s (N-H), 1680s (C=O), 1620m (C=C). ^1^H-NMR (300 MHz, CDCl_3_/DMSO-*d*_6_): δ (ppm) 11.4 (s, 1H, 1 × pyrrolyl H), 8.93–9.10 (m, 2H, 2 × naphthyl H), 8.06–8.26 (m, 3H, 3 × naphthyl H), 7.90–7.93 (m, 2H, 2 × Ph H), 7.79–7.81 (m, 1H, 1 × Ph H), 7.69–7.75 (m, 2H, 2 × Ph H), 7.49–7.59 (m, 2H, 2 × naphthyl H), 7.13–7.21 (br, 1H, 1 × pyrrolyl H), 6.90–7.02 (m, 1H, 1 × pyrrolyl H). ^13^C-NMR (75.5 MHz, CDCl_3_/DMSO-*d*_6_): δ (ppm) 173.0, 130.5, 128.0, 127.3, 127.2, 126.8, 126.4, 126.0, 125.7, 125.0, 124.7, 124.6, 119.7. Anal. C, H, N. (C_21_H_15_NO); Calc. % C: 75.22, H: 4.32, N: 4.62; found % C: 75.49, H: 4.63, N: 4.58.

*(4-Chlorophenyl)[4-(naphth-1-yl)-1*H*-pyrrol-3-yl]methanone* (**2v**): The general method was followed using chalcone **1v**. IR (Nujol) (ν cm^−1^): 3200w (N-H), 3090m (Ar-H), 1700s (C=O). ^1^H-NMR (300 MHz, CDCl_3_): δ (ppm) 8.76–8.96 (m, 2H, 2 × naphthyl H), 8.22–8.28 (m, 1H, 1 × naphthyl H), 8.06–8.10 (br, 1H, 1 × naphthyl H), 7.86–7.96 (m, 3H, 2 × Ph H, 1 × naphthyl H), 7.6–7.53 (m, 4H, 2 × Ph H, 2 × naphthyl H), 7.08–7.11 (m, 1H, 1 × pyrrolyl H), 6.95-6.99 (m, 1H, 1 × pyrrolyl H). ^13^C-NMR (75.5 MHz, CDCl_3_): δ (ppm) 174.0, 145.0, 141.6, 128.0, 127.0, 124.8, 122.8, 112.0, 107.4, 104.7, 100.4. Anal. C, H, N. (C_21_H_14_ClNO); Calc. % C: 74.64, H: 3.96, N: 4.58; found % C: 74.82, H: 4.08, N: 4.19.

*[4-(1H-Indol-5-yl)-1H-pyrrol-3-yl](phenyl)methanone* (**2vi**): The general method was followed using chalcone **1vi**. IR (Nujol) (ν cm^−1^): 3200m (N-H), 1710s (C=O). ^1^H-NMR (300 MHz, DMSO-*d*_6_): δ (ppm) 10.97 (s, 1H, 1 × indolyl H), 10.22 (s, 1H, 1 × pyrrolyl H), 7.99–8.03 (br s, 1H, 1 × indolyl H), 7.56–7.73 (m, 7H, 7 × indolyl/Ph H), 7.06–7.24 (m, 3H, 2 × pyrrolyl H, 1 × indolyl H), 6.80–6.81 (br s, 1H, 1 × indolyl H). ^13^C-NMR (75.5 MHz, DMSO-*d*_6_): δ (ppm) 192.0, 142.0, 135.0, 131.0, 129.0, 128.0, 127.0, 126.0, 125.0, 124.0, 122.0, 120.0, 117.0, 114.0, 110.0. Anal. C, H, N. (C_19_H_14_N_2_O); Calc. % C: 79.70, H: 4.93, N: 4.89; found %: C: 79.73, H: 5.19, N: 4.85.

### 3.3. Physicochemical Studies

Reverse-phase thin layer chromatography (RPTLC) was used for the determination of R_M_ values. Silica gel plates were saturated with 5% *v*/*v* liquid paraffin in petroleum ether, and the mobile phase was methanol/water (75/25 *v*/*v*) with the addition of ammonia drops. Spots were detected under UV light. R_M_ values were determined by the corresponding R_f_ values (from five measurements for each compound) using the equation [43]:

R_M_ = log[(1/R_f_) − 1] (1)

Lipophilicity was theoretically calculated as Clog *P* values using the program of Biobyte Corporation [45]. For the calculation of physicochemical properties of the pyrrolyl derivatives, the Spartan program v.5.1.3 (Wavefunction Inc.) was used. Briefly, for each compound: (1) the structure was optimized with the quantum mechanics semi-empirical method AM1 (Austin Model 1); and (2) conformers were randomly reproduced with the Monte Carlo method by selecting all possible bonds that could rotate and by applying the dynamic field of molecular mechanics, Merck (MMFF94). Calculations were performed on the conformer with the minimum energy using quantum mechanics methods in level 6-31G* (and for compounds bearing bromium: 3-21G*) and semi-empirical in level AM1-SM2.

The C-QSAR program [45] was also used for the calculation of the values of overall molar refractivity (CMR) and of the molar refractivity of substituents (MR).

### 3.4. Biological Assays

For the *in vitro* biological assays, the tested compounds, as stock solutions (10 mM), were dissolved in DMSO. Final solutions of several concentrations were prepared for the assays given below. The results were the mean of at least two different experiments, and the standard deviation did not exceed 10%.

#### 3.4.1. Biological Assays *in Vitro*

##### Interaction with the Stable Free Radical 2,2-Diphenyl-1-picrylhydrazyl

To a solution of DPPH in absolute ethanol (50 μΜ final concentration), the appropriate volume of the compounds (final concentrations of 100 and 1000 μΜ) dissolved in DMSO was added. The absorbance was measured at 517 nm after 20 and 60 min at room temperature [52].

##### Superoxide Radical Scavenging Activity

The superoxide anion radicals were produced non-enzymatically by mixing phenazine methosulfate (PMS) with NADH and air-oxygen. The production of superoxide radicals was estimated with the nitroblue-tetrazolium (NBT) method with 3 μΜ PMS, 78 μΜ NADH and 25 μΜ NBT in 19 μΜ phosphate buffer pH 7.4. The absorbance was measured at 560 nm at room temperature. The tested compounds were added in the mixture 2 min before the addition of NADH [52].

##### Inhibition of Heme-Dependent Lipid Peroxidation of Linoleic Acid

Firstly, 0.4 mL of heme (50 μΜ), 0.25 mL of sodium linoleate (400 μΜ), 0.1 mL of the solutions of the tested compounds (concentrations varying from 10–100 μΜ in DMSO) and 0.25 mL of H_2_O_2_ (500 μΜ) were incubated for 10 min in 37 °C in a phosphate buffer (KH_2_PO_4_–KOH 50 mM, pH 7.4). The peroxidation products were identified with the reaction of thiobarbituric acid, by adding successively 0.5 mL of 40% trichloroacetic acid (TCA) and 0.5 mL of 2% of thiobarbituric acid (TBA) [52]. The addition was followed by stirring and heating at 90 °C for 20 min, and then, 1 mL of acetic acid and 1 mL of chloroform were added and the absorbance measured at 535 nm. IC_50_ values were determined.

##### Inhibition of Soybean Lipoxygenase

The tested compounds were dissolved in DMSO, and the final concentration was 100 μΜ. The compounds were incubated at room temperature with sodium linoleate as a substrate (0.1 mL) in a buffer solution of Tris:HCl (pH 9.00) and 0.2 mL of a solution of soybean lipoxygenase [52]. The conversion of linoleic acid into 13-hydroperoxylinoleic acid was measured at 234 nm and was compared to the reference compound nordihydroguaiaretic acid (NDGA).

##### Inhibition of Cycloxygenase-2 (COX-2)

COX-2 inhibitory activity was determined by using arachidonic acid (AA) as a substrate in buffer (0.1 M Tris pH 8.5) and TMPD as a co-substrate. Ten microliters of the DMSO solution of the tested compounds (final concentration 100 μM) were added in a buffer (Tris-HCl pH 8.5), followed by the addition of 0.75 μΜ heme, 80 μΜ arachidonic acid, COX-2 and 128 μΜ TMPD. The mixture was incubated for 5 min at 37 °C, and after vigorous shaking, the absorbance was measured at 590 nm [52,53].

##### Inhibition of Interleukin-6 (IL-6) (Interleukin-6 (Mouse))

The method was based on an immunometric double antibody sandwich technique. Each well of the microwell plate was coated with a specific for mouse IL-6 rat monoclonal antibody. Tested compounds at 10 μΜ and standards were incubated in the wells, and after rinsing, a second monoclonal antibody was added, which was conjugated with biotin and detected the captured IL-6. The recognition of double antibody sandwiches was done with HRP-conjugated streptavidin. The concentration of IL-6 was determined by measuring the enzymatic activity of HRP using the chromogenic substrate TMB (3,3′,5,5′-tetramethylbenzidine). The absorbance of the reaction product was measured at 450 nm, and the intensity of the color was directly proportional to the amount of bound HRP-streptavidin conjugate, which was proportional to the concentration of IL-6.

##### Inhibition of the Proteolytic Activity of Trypsin

The tested compounds (concentrations varying from 10 μΜ–100 μΜ in DMSO) were dissolved in a phosphate buffer (0.1 M K_2_HPO_4_–NaH_2_PO_4_·2H_2_O, pH = 7.6), and 0.2 mL of trypsin 0.075 mg/mL were added. The mixture was incubated at room temperature for 20 min, and then, 1 mL of bovine albumin (6 g/100 mL) was added. The mixture was incubated at 37 °C for 30 min. The reaction was stopped with the addition of 1 mL of trichloroacetic acid (5% *w*/*w*) Separation of the phases followed, with measurement of the supernatants at 280 nm at room temperature [54]. IC_50_ values were determined.

#### 3.4.2. Biological Assay *in Vivo*

##### Inhibition of Rat Paw Edema Induced by Carrageenan

Edema was induced in the right hind paw of Fischer 344 rats (150–200 g). In the study, both males and females were included, with the exemption of pregnant females. The animals, which have been bred in our laboratory, were housed under standard conditions and received a diet of commercial food pellets and water *ad libitum* during maintenance. One hour before the onset of the experiment, food and water were removed. The tested compounds (dose 0.01 mmol/kg body weight) were diluted in water with a few drops of Tween 80, and they were administered intraperitoneally simultaneously with the intradermal injection of 0.1 mL 2% of carrageenan in water. The animals were euthanatized 3.5 h after the carrageenan administration. The difference between the weight of injected and uninjected paws was calculated for each animal and was compared to control animals, which were treated with water [52]. Indomethacin was used as a reference compound in 0.01 mmol/kg. The results were expressed as the percent inhibition of the edema (CPE %).

## 4. Conclusions

In the present study, six pyrrolyl derivatives were designed and synthesized using chalcones as the starting material. Lipophilicity as R_M_ values was determined, and physicochemical properties were calculated. The biological activity of the pyrrolyl derivatives was evaluated in antioxidant and anti-inflammatory assays. Compound **2iii** was found to be a potent selective COX-1 inhibitor, and as mentioned in recent literature, such compounds could be useful in neurodegenerative diseases, including Parkinson’s and Alzheimer’s disease, in which the production of prostaglandins in neurons and neuroglia is increased. Compound **2v** presented satisfactory anti-inflammatory activity, inhibiting COX-2, IL-6 and trypsin. The presence of an electron-acceptor substituent (Cl) on the phenyl ring on Ar^2^ and the increase of the volume of Ar^2^ simultaneously improve the anti-proteolytic activity significantly. A carbonyl group into the vicinal diaryl substitution in 3,4-positions of the pyrrolyl ring was found to be the structural feature for the pharmacophore. The presented combination of biological activities of **2v** support that this pyrrolyl derivative is a promising pleiotropic bioactive molecule. Additional studies of the physicochemical properties could lead to useful conclusions for the future design of compounds with the ability to interact with multiple biological targets. Further investigation is in progress.

## Abbreviations

AA: arachidonic acid; Clog *P*: theoretically-calculated lipophilicity; CMR: calculated molar refractivity; CNS: central nervous system; COX: cyclooxygenase; CPE: carrageenan-induced paw edema; DPPH: 2,2-diphenyl-1-picrylhydrazyl; HIV: human immunodeficiency virus; HMG-CoA reductase: 3-hydroxy-3-methyl-glutaryl coenzyme A reductase; HRP-streptavidin: horseradish peroxidase-labeled streptavidin; IL-6: interleukin-6; IMA: indomethacin; LOX: lipoxygenase; LP: lipid peroxidation; LTB4: leukotriene B4; MR: molar refractivity of substituent; NADH: nicotinamide adenine dinucleotide; NBT: nitroblue-tetrazolium; NDGA: nordihydroguaiaretic acid; NSAIDs: non-steroid anti-inflammatory drugs; PMS: phenazine methosulfate; RA: reducing activity; RPTLC: reverse-phase thin layer chromatography; TBA: thiobarbituric acid; TCA: trichloroacetic acid; TMB: 3,3′,5,5′-tetramethylbenzidine; TMPD: *N*,*N*,*N′*,*N′*-tetramethyl-*p*-phenylenediamine; TosMIC: *p*-toluenesulfonylmethyl isocyanide.

## References

[1] Salzano S., Checconi P., Hanschmann E.M., Lillig C.H., Bowler L.D., Chan P., Vaudry D., Mengozzi M., Coppo L., Sacre S. (2014). Linkage of inflammation and oxidative stress via release of glutathionylated peroxiredoxin-2, which acts as a danger signal. Proc. Natl. Acad. Sci. USA.

[2] Pizza V., Agresta A., D’Acunto C.W., Festa M., Capasso A. (2011). Neuroinflamm-aging and neurodegenerative diseases: An overview. CNS Neurol. Disord. Drug Targets.

[3] Sethi G., Shanmugam M.K., Ramachandran L., Kumar A.P., Tergaonkar V. (2012). Multifaceted link between cancer and inflammation. Biosci. Rep..

[4] Aggarwal B.B., Vijayalekshmi R.V., Sung B. (2009). Targeting inflammatory pathways for prevention and therapy of cancer: Short-term friend, long-term foe. Clin. Cancer Res..

[5] Charlier C., Michaux C. (2003). Dual inhibition of cyclooxygenase-2 (COX-2) and 5-lipoxygenase (5-LOX) as a new strategy to provide safer non-steroidal anti-inflammatory drugs. Eur. J. Med. Chem..

[6] Botting R.M. (2000). Mechanism of action of acetaminophen: Is there a cycloxygenase 3?. Clin. Infect. Dis..

[7] Vane J.R., Bakhle Y.S., Botting R.M. (1998). Cyclooxygenases 1 and 2. Annu. Rev. Pharmacol. Toxicol..

[8] Atukorala I., Hunter D.J. (2013). Valdecoxib: The rise and fall of a COX-2 inhibitor. Expert Opin. Pharmacother..

[9] Marwali M.R., Mehta J.L. (2006). COX-2 inhibitors and cardiovascular risk. Inferences based on biology and clinical studies. Thromb. Haemost..

[10] Gilroy D.W., Tomlinson A., Willoughby D.A. (1998). Differential effects of inhibitors of cyclooxygenase (cyclooxygenase 1 and cyclooxygenase 2) in acute inflammation. Eur. J. Pharmacol..

[11] Leone S., Ottani A., Bertolini A. (2007). Dual acting anti-inflammatory drugs. Curr. Top. Med. Chem..

[12] Aïd S., Bosetti F. (2011). Targeting cyclooxygenases-1 and -2 in neuroinflammation: Therapeutic implications. Biochimie.

[13] Choi S.H., Aid S., Bosetti F. (2009). The distinct roles of cyclooxygenase-1 and -2 in neuroinflammation: Implications for translational research. Trends Pharmacol. Sci..

[14] Liedtke A.J., Crews B.C., Daniel C.M., Blobaum A.L., Kingsley P.J., Ghebreselasie K., Marnett L.J. (2012). Cyclooxygenase-1-selective inhibitors based on the (*E*)-2′-des-methyl-sulindac sulfide scaffold. J. Med. Chem..

[15] Khan S.A., Asiri A.M., Alamry K.A., El-Daly S.A., Zayed M.A. (2013). Eco-friendly synthesis and *in vitro* antibacterial activities of some novel chalcones. Bioorg. Khim..

[16] Łącka I., Konieczny M.T., Bułakowska A., Rzymowski T., Milewski S. (2011). Antifungal action of the oxathiolone-fused chalcone derivative. Mycoses.

[17] De León E.J., Alcaraz M.J., Dominguez J.N., Charris J., Terencio M.C. (2003). 1-(2,3,4-Trimethoxyphenyl)-3-(3-(2-chloroquinolinyl))-2-propen-1-one, a chalcone with analgesic, anti-inflammatory and immunomodulatory properties. Inflamm. Res..

[18] Bukhari S.N., Jantan I., Jasamai M. (2013). Anti-inflammatory trends of 1,3-diphenyl-2-propen-1-one derivatives. Mini Rev. Med. Chem..

[19] Kamal A., Srinivasulu V., Nayak V.L., Sathish M., Shankaraiah N., Bagul C., Reddy N.V., Rangaraj N., Nagesh N. (2014). Design and synthesis of C3-pyrazole/chalcone-linked beta-carboline hybrids: Antitopoisomerase I, DNA-Interactive and apoptosis-inducing anticancer agents. ChemMedChem.

[20] Kumar C.S., Loh W.S., Ooi C.W., Quah C.K., Fun H.K. (2013). Structural correlation of some heterocyclic chalcone analogues and evaluation of their antioxidant potential. Molecules.

[21] Luo Y., Song R., Li Y., Zhang S., Liu Z.J., Fu J., Zhu H.L. (2012). Design, synthesis, and biological evaluation of chalcone oxime derivatives as potential immunosuppressive agents. Bioorg. Med. Chem. Lett..

[22] Okunrobo L.O., Usifoh C.O., Uwaya J.O. (2006). Anti-inflammatory and gastroprotective properties of some chalcones. Acta Pol. Pharm..

[23] Sharma H., Patil S., Sanchez T.W., Neamati N., Schinazi R.F., Buolamwini J.K. (2011). Synthesis, biological evaluation and 3D-QSAR studies of 3-keto salicylic acid chalcones and related amides as novel HIV-1 integrase inhibitors. Bioorg. Med. Chem..

[24] Nielsen S.F., Christensen S.B., Cruciani G., Kharazmi A., Liljefors T. (1998). Antileishmanial chalcones: Statistical design, synthesis and three-dimensional structure–activity relationship analysis. J. Med. Chem..

[25] Murugesan D., Kaiser M., White K.L., Norval S., Riley J., Wyatt P.G., Charman S.A., Read K.D., Yeates C., Gilbert I.H. (2013). Structure-activity relationship studies of pyrrolone antimalarial agents. ChemMedChem.

[26] Mohamed M.S., Kamel R., Fatahala S.S. (2010). Synthesis and biological evaluation of some thio containing pyrrolo[2,3-*d*]pyrimidine derivatives for their anti-inflammatory and anti-microbial activities. Eur. J. Med. Chem..

[27] Kim K.J., Choi M.J., Shin J.S., Kim M., Choi H.E., Kang S.M., Jin J.H., Lee K.T., Lee J.Y. (2014). Synthesis, biological evaluation, and docking analysis of a novel family of 1-methyl-1*H*-pyrrole-2,5-diones as highly potent and selective cyclooxygenase-2 (COX-2) inhibitors. Bioorg. Med. Chem. Lett..

[28] Arumugam N., Raghunathan R., Almansour A.I., Karama U. (2012). An efficient synthesis of highly functionalized novel chromeno[4,3-*b*]pyrroles and indolizino[6,7-*b*]indoles as potent antimicrobial and antioxidant agents. Bioorg. Med. Chem. Lett..

[29] Wang Y., Lu H., Zhu Q., Jiang S., Liao Y. (2010). Structure-based design, synthesis and biological evaluation of new *N*-carboxyphenylpyrrole derivatives as HIV fusion inhibitors targeting gp41. Bioorg. Med. Chem. Lett..

[30] Xie L., Xiao Y., Wang F., Xu Y., Qian X., Zhang R., Cui J., Liu J. (2009). Novel acenaphtho[1,2-*b*]pyrrole-carboxylic acid family: synthesis, cytotoxicity, DNA-binding and cell cycle evaluation. Bioorg. Med. Chem..

[31] Caruso M., Valsasina B., Ballinari D., Bertrand J., Brasca M.G., Caldarelli M., Cappella P., Fiorentini F., Gianellini L.M., Scolaro A. (2012). 5-(2-amino-pyrimidin-4-yl)-1*H*-pyrrole and 2-(2-amino-pyrimidin-4-yl)-1,5,6,7-tetrahydro-pyrrolo[3,2-*c*]pyridin-4-one derivatives as new classes of selective and orally available Polo-like kinase 1 inhibitors. Bioorg. Med. Chem. Lett..

[32] Weagle G., Gupta A., Bérubé G., Chapados C. (2010). Evaluation of *in vivo* biological activities of tetrapyrrole ethanolamides as novel anticancer agents. J. Photochem. Photobiol. B..

[33] Ji X., Su M., Wang J., Deng G., Deng S., Li Z., Tang C., Li J., Li J., Zhao L., Jiang H., Liu H. (2014). Design, synthesis and biological evaluation of hetero-aromatic moieties substituted pyrrole-2-carbonitrile derivatives as dipeptidyl peptidase IV inhibitors. Eur. J. Med. Chem..

[34] Nishida H., Hasuoka A., Arikawa Y., Kurasawa O., Hirase K., Inatomi N., Hori Y., Sato F., Tarui N., Imanishi A. (2012). Discovery, synthesis, and biological evaluation of novel pyrrole derivatives as highly selective potassium-competitive acid blockers. Bioorg. Med. Chem..

[35] Bratton L.D., Auerbach B., Choi C., Dillon L., Hanselman J.C., Larsen S.D., Lu G., Olsen K., Pfefferkorn J.A., Robertson A. (2007). Discovery of pyrrole-based hepatoselective ligands as potent inhibitors of HMG-CoA reductase. Bioorg. Med. Chem..

[36] Dannhardt G., Kiefer W., Krämer G., Maehrlein S., Nowe U., Fiebich B. (2000). The pyrrole moiety as a template for COX-1/COX-2 inhibitors. Eur. J. Med. Chem..

[37] Kouskoura M., Hadjipavlou-Litina D., Giakoumakou M. (2008). Synthesis and AntiInflammatory Activity of Chalcones and Related Mannich Bases. Med. Chem..

[38] Artico M., Di Santo R., Costi R., Massa S., Retico A., Artico M., Apuzzo G., Simonetti G., Strippoli V. (1995). Antifungal agents. 9. 3-Aryl-4-[α-(1*H*-imidazol-1-yl)arylmethyl]pyrroles: A new class of potent anti-Candida agents. J. Med. Chem..

[39] Jones J.E. (1958). Supersensitization of Photographic Emulsions. US Patent.

[40] Chiaradia L.D., Mascarello A., Purificação M., Vernal J., Cordeiro M.N., Zenteno M.E., Villarino A., Nunes R.J., Yunes R.A., Terenzi H. (2008). Synthetic chalcones as efficient inhibitors of Mycobacterium tuberculosis protein tyrosine phosphatase PtpA. Bioorg. Med. Chem. Lett..

[41] Stroba A., Schaeffer F., Hindie V., Lopez-Garcia L., Adrian I., Fröhner W., Hartman R.W., Biondi R.M., Engel M. (2009). 3,5-Diphenylpent-2-enoic acids as allosteric activators of the protein kinase PDK1: Structure-activity relationships and thermodynamic characterization of binding as paradigms for PIF-binding pocket-targeting compound. J. Med. Chem..

[42] Cocconcelli G., Diodato E., Caricasole A., Gaviraghi G., Genesio E., Ghiron C., Magnoni L., Pecchioli E., Plazzi P.V., Terstappen G.C. (2008). Aryl azoles with neuroprotective activity-parallel synthesis and attempts at target identification. Bioorg. Med. Chem..

[43] Rekker R. (1977). Hydrophobic Fragmental Constant.

[44] Hansch C., Leo A.J., Heller S.R. (1995). Exploring QSAR Fundamentals and Applications in Chemistry and Biolorgy.

[B45-molecules-20-16354] 45.BioByte Corporation, C-QSAR database, 201 W Fourth str. Suite # 204, Claremont, CA 91711–4707, USA.

[46] Flohe L., Beckman R., Giertz H., Loschen G., Sies H. (1985). Oxygen-centered free radicals as mediators of inflammation. Oxidative Stress.

[47] Niki E. (2012). Do antioxidants impair signaling by reactive oxygen species and lipid oxidation products?. FEBS Lett..

[48] Evans J.P., Cecchini R., Halliwell B. (1992). Oxidative damage to lipids and alpha 1-antiproteinase by phenylbutazone in the presence of haem proteins: Protection by ascorbic acid. Biochem. Pharmacol..

[49] Crooks S.W., Stockley R.A. (1998). Leukotriene B4. Int. J. Biochem. Cell. Biol..

[50] Peperidou A., Kapoukranidou D., Kontogiorgis C., Hadjipavlou-Litina D. (2014). Multitarget Molecular Hybrids of Cinnamic Acids. Molecules.

[51] Demopoulos V.J., Rekka E. (1995). Isomeric benzoylpyrroloacetic acids: Some structural aspects for aldose reductase inhibitory and anti-inflammatory activities. J. Pharm. Sci..

[52] Pontiki E., Hadjipavlou-Litina D. (2006). Antioxidant and anti-inflammatory activity of aryl-acetic and hydroxamic acids as novel lipoxygenase inhibitors. Med. Chem..

[53] Kulmacz R.J., Lands W.E.M. (1983). Requirements for hydroperoxide by the cyclooxygenase and peroxidase activities of prostaglandin H synthase. Prostaglandins.

[54] Michaelidou A., Hadjipavlou-Litina D., Matsini I., Tsitsogianni E. (2007). Heterocyclic aryl(phenyl)acetic acid and aryl acetohydroxamic acids as antiinflammatory-antioxidant agents and inhibitors of lipoxygenase and serine proteases. Med. Chem..

